# Supplementary material for: Empirical methods for controlling false positives and estimating confidence in ChIP-Seq peaks
Source: BMC Bioinformatics. 2008 Dec 5;9:523. doi: 10.1186/1471-2105-9-523 (PMC2628906; doi:10.1186/1471-2105-9-523)
Supplement: Additional File 1 — A variety of html documents from the USeq web site detailing the available applications, their best usage, output file type descriptions, command line menus, etc. [file 1471-2105-9-523-S1.zip › USeqUserGuides/JavaDoc.zip!/JavaDoc/meme/MotifSearchResult.html]

MotifSearchResult


|  |  |  |
| --- | --- | --- |
| **Overview** \| **Package** \| **Class** \| **Use** \| **Tree** \| **Deprecated** \| **Index** \| **Help** | |  |
| **PREV CLASS**   **NEXT CLASS** | **FRAMES**    **NO FRAMES**     **All Classes** |  |
| SUMMARY: NESTED \| FIELD \| CONSTR \| METHOD | DETAIL: FIELD \| CONSTR \| METHOD |  |


---


## meme Class MotifSearchResult

```
java.lang.Object
  meme.MotifSearchResult
```

**All Implemented Interfaces:**: java.lang.Comparable

---

``` public class MotifSearchResult extends java.lang.Object implements java.lang.Comparable ```

Holds the results of searching a multi-fasta seq file with a particular motif.

---

**Constructor Summary**

`MotifSearchResult(MemeMotif mm,
java.lang.String file,
int numberOfSeqsSearched)`


| **Method Summary** | |
| --- | --- |
| `int` | `compareTo(java.lang.Object otherObject)` |
| `double` | `getAddOneLLPSPMCutOffScore()` |
| `int` | `getFileNumber()` |
| `java.util.ArrayList` | `getHitsAddOneLLPSPM()` |
| `java.util.ArrayList` | `getHitsMemeLLPSPM()` |
| `double` | `getMemeLLPSPMCutOffScore()` |
| `MemeMotif` | `getMemeMotif()` |
| `double` | `getMotifSig()` |
| `int` | `getNumHitsAddOneLLPSPM()` |
| `int` | `getNumHitsMemeLLPSPM()` |
| `int` | `getnumSeqsSearched()` |
| `java.lang.String` | `getSearchedSeqFile()` |
| `void` | `setAddOneLLPSPMCutOffScore(double data)` |
| `void` | `setFileNumber(int data)` |
| `void` | `setHitsAddOneLLPSPM(java.util.ArrayList data)` |
| `void` | `setHitsMemeLLPSPM(java.util.ArrayList data)` |
| `void` | `setMemeLLPSPMCutOffScore(double data)` |
| `void` | `setMotifSig(double data)` |
| `void` | `setNumHitsAddOneLLPSPM(int data)` |
| `void` | `setNumHitsMemeLLPSPM(int data)` |

**Methods inherited from class java.lang.Object**

`equals, getClass, hashCode, notify, notifyAll, toString, wait, wait, wait`

**Constructor Detail**

### MotifSearchResult

```
public MotifSearchResult(MemeMotif mm,
                         java.lang.String file,
                         int numberOfSeqsSearched)
```


**Method Detail**

### compareTo

```
public int compareTo(java.lang.Object otherObject)
```

:   **Specified by:**: `compareTo` in interface `java.lang.Comparable`

---


### setMotifSig

```
public void setMotifSig(double data)
```

---


### setFileNumber

```
public void setFileNumber(int data)
```

---


### setMemeLLPSPMCutOffScore

```
public void setMemeLLPSPMCutOffScore(double data)
```

---


### setAddOneLLPSPMCutOffScore

```
public void setAddOneLLPSPMCutOffScore(double data)
```

---


### setHitsMemeLLPSPM

```
public void setHitsMemeLLPSPM(java.util.ArrayList data)
```

---


### setHitsAddOneLLPSPM

```
public void setHitsAddOneLLPSPM(java.util.ArrayList data)
```

---


### setNumHitsMemeLLPSPM

```
public void setNumHitsMemeLLPSPM(int data)
```

---


### setNumHitsAddOneLLPSPM

```
public void setNumHitsAddOneLLPSPM(int data)
```

---


### getFileNumber

```
public int getFileNumber()
```

---


### getMotifSig

```
public double getMotifSig()
```

---


### getMemeMotif

```
public MemeMotif getMemeMotif()
```

---


### getMemeLLPSPMCutOffScore

```
public double getMemeLLPSPMCutOffScore()
```

---


### getAddOneLLPSPMCutOffScore

```
public double getAddOneLLPSPMCutOffScore()
```

---


### getSearchedSeqFile

```
public java.lang.String getSearchedSeqFile()
```

---


### getHitsMemeLLPSPM

```
public java.util.ArrayList getHitsMemeLLPSPM()
```

---


### getHitsAddOneLLPSPM

```
public java.util.ArrayList getHitsAddOneLLPSPM()
```

---


### getNumHitsMemeLLPSPM

```
public int getNumHitsMemeLLPSPM()
```

---


### getNumHitsAddOneLLPSPM

```
public int getNumHitsAddOneLLPSPM()
```

---


### getnumSeqsSearched

```
public int getnumSeqsSearched()
```


---


|  |  |  |
| --- | --- | --- |
| **Overview** \| **Package** \| **Class** \| **Use** \| **Tree** \| **Deprecated** \| **Index** \| **Help** | |  |
| **PREV CLASS**   **NEXT CLASS** | **FRAMES**    **NO FRAMES**     **All Classes** |  |
| SUMMARY: NESTED \| FIELD \| CONSTR \| METHOD | DETAIL: FIELD \| CONSTR \| METHOD |  |


---
